# Rapid GC-MS Characterization of Oleoresin, Turpentine and Rosin Using Tailored Chromatographic Programs

**DOI:** 10.3390/ijms27041690

**Published:** 2026-02-09

**Authors:** Nalin Seixas, Sónia A. O. Santos, Armando J. D. Silvestre

**Affiliations:** CICECO—Aveiro Institute of Materials, Department of Chemistry, University of Aveiro, 3810-193 Aveiro, Portugal; santos.sonia@ua.pt (S.A.O.S.); armsil@ua.pt (A.J.D.S.)

**Keywords:** gas chromatography, mass spectrometry, oleoresin, rosin, turpentine

## Abstract

Oleoresin from *Pinus* spp. consists of turpentine and rosin, whose compositional variability demands reliable analytical methods for quality control and industrial processing. This study provides three rapid methods for qualitative and quantitative analyses of oleoresin, turpentine, and rosin by gas chromatography coupled with mass spectrometry (GC-MS) using a single DB-1 column and matrix-specific temperature programs. Oleoresin and rosin were first derivatized using diazomethane, and compounds were identified by elution order, fragmentation patterns, and reference mass spectra. Quantification employed external calibration with α-pinene and abietic acid as representative standards. In *P. pinaster* oleoresin, the main terpenic compounds were α-pinene (6.67 ± 1.08%), longifolene (2.45 ± 0.20%), and β-caryophyllene (1.71 ± 0.15%), while levopimaric (33.75 ± 2.70%), neoabietic (13.97 ± 1.70%), and abietic acids (12.60 ± 2.90%) predominated among resin acids. *P. elliottii* rosin contained mainly abietic (45.99 ± 4.82%), isopimaric (16.95 ± 2.55%), and palustric acids (9.74 ± 1.20%), and its turpentine comprised mainly α-pinene (34.16 ± 2.45%) and β-pinene (30.03 ± 1.20%). This unified GC–MS framework, supported by representative calibration standards, enables identification of >95% of compounds in pine matrices. Furthermore, once compound identification has been established through GC-MS, GC coupled with flame ionization detector (GC-FID) can be employed for routine quantitative analysis.

## 1. Introduction

**Figure 1:**
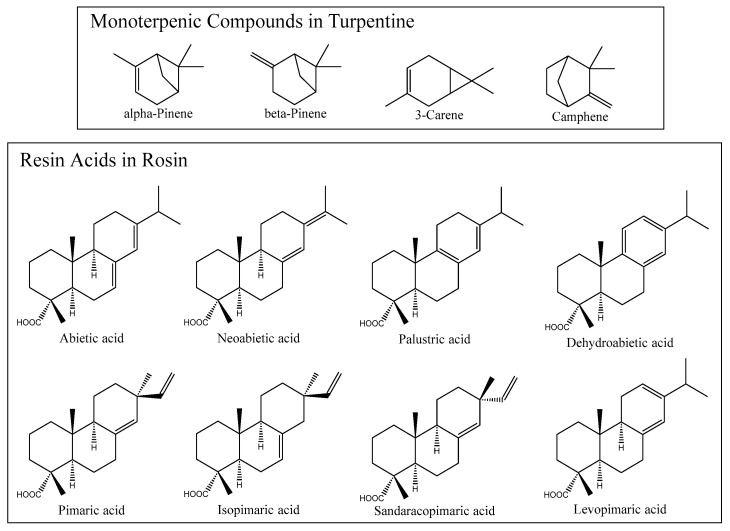
Representative chemical structures of major monoterpenic compounds and resin acids identified in turpentine and rosin, respectively [2,7,8].

Natural resin or oleoresin is a viscous exudate obtained from pine trees (*Pinus* spp.), particularly *P. elliottii* (slash pine) and *P. pinaster* (maritime pine) [1,2]. Oleoresin consists of two main fractions, a volatile one, known as turpentine, and a non-volatile solid fraction, known as rosin or colophony. These two products are separated by distillation, during which turpentine evaporates, leaving rosin behind as a solid residue [1,2]. Turpentine is mainly composed of terpenic compounds, particularly monoterpenic compounds, with α-pinene, β-pinene, camphene, and 3-carene being the most frequently identified constituents (Figure 1). Turpentine is widely used as a solvent and as a raw material in the synthesis of fragrances, flavors, and fine chemicals [3,4].

Rosin, on the other hand, is a mixture of monocarboxylic diterpenic compounds, also known as resin acids, and neutral diterpenic compounds, mainly aldehydes and alcohols [1,2,5,6]. Its unique characteristics make it widely applicable in the production of numerous products, such as adhesives, varnishes, printing inks, and pharmaceutical products [7,8,9,10]. Abietic-type and pimaric-type resin acids represent the predominant compounds typically identified in rosin [1,2,6]. Abietic, palustric, levopimaric, and neoabietic acids are among the most common abietic-type resin acids characterized by a conjugated double bond system, whereas isopimaric and pimaric acids represent the pimaric-type resin acids bearing nonconjugated double bonds. Dehydroabietic acid, although structurally related to the abietane family, is distinguished by the presence of an aromatic ring and the absence of a conjugated double bond (Figure 1) [5,7,8].

The changes in composition and amount of each terpenic compound and resin acid in oleoresin, turpentine, and rosin depend on the pine tree species, geographical origin, seasonal factors, and resin collection method [11,12,13,14]. Moreover, chemical complexity, especially among the resin acids, arises not only from the diversity of chemical structures but also from their stereoisomerism (mostly structural isomers) [6,7,8]. Because of this variability in chemical composition, analytical characterization is critically important, particularly at the industrial level, for quality control and subsequent product processing, as even small changes in terpenic compound and resin acid profiles can influence the physicochemical and functional characteristics of the final materials. In addition, post-processing chemical analysis ensures compliance with established specifications [6,8].

Gas chromatography coupled with mass spectrometry (GC-MS) is a powerful analytical tool for the chemical analysis of complex organic mixtures such as oleoresin, turpentine, and rosin [15,16]. In 1959, Hudy described the first method using GC-MS for the identification of resin acid methyl esters [17,18]. Subsequently, several studies reported the characterization of oleoresin, rosin or specific resin acids using GC-MS, generally in the form of methyl or trimethylsilyl esters, since compounds containing carboxylic acids require derivatization before they can be analyzed by GC-MS due to their low volatility [16,19,20,21,22,23,24,25,26,27]. Among various derivatization techniques, quaternary ammonium hydroxides, and particularly tetramethylammonium hydroxide (TMAH), are commonly employed to pyrolytically generate methyl esters in the GC injector due to their simplicity and straightforward application, even at the industrial level [28,29,30,31]. Nevertheless, this method may result in the formation of multiple derivatives from a single compound, producing complex mixtures that require careful interpretation [28,29,30,31]. Moreover, the full conversion of free acids into methyl esters is hard to control unless a large excess of the derivatizing reagent and high temperatures are used, which also lead to significant injector contamination and, in general, only enables relative composition determination approaches (based on area percentages) [28,29,30,31].

The most effective approach to quantitatively converting resin acids into methyl esters involves the use of diazomethane. Diazomethane is a gaseous and unstable substance, but it can be safely synthesized in situ from *N*-methyl-*N*-(*p*-tolylsulfonyl)nitrosamide in the presence of a base and used directly for methylation in a closed system [32,33].

Currently, GC-MS analyses of oleoresin, rosin, and turpentine rely mostly on relative peak area percentages, rather than absolute quantification using external calibration [25,28,32]. This can distort real concentrations, especially when undetected compounds are neglected, or the detector response varies among analytes with different volatility or ionization efficiency, making relative quantification less reliable [25,28,32]. While achieving absolute calibration may be challenging due to limited availability of pure standards, high costs, and the structural similarity among sample compounds, particularly among monoterpenic compounds and resin acids, quantification accuracy can be substantially improved by employing structurally similar compounds as representative standards for the analysis of each compound family.

Moreover, most existing studies focus on the analysis of a single matrix, rather than developing methods applicable to all oleoresin components. Therefore, to the best of our knowledge, this is the first study combining diazomethane methylation with a single non-polar GC column and using only temperature program adjustments to enable the identification and accurate quantification of compounds present in oleoresin, turpentine, and rosin from two different types of pine trees (*P. pinaster* and *P. elliottii*). This approach could provide a simple and transferable workflow that can be readily implemented in industrial quality control laboratories with minimal modifications to existing infrastructures.

## 2. Results and Discussion

Given the chemical diversity of *Pinus* spp. oleoresins, it is essential to employ distinct analytical methods to accurately characterize the complete oleoresin as well as its isolated fractions (turpentine and rosin). Accordingly, this study developed three GC-MS temperature programs, each specifically designed for the unique properties of each pine matrix type, all using the same nonpolar DB-1 GC column. This strategy minimizes instrumental complexity and enables rapid, interchangeable analysis across all three pine matrices without column changes or extensive reconfiguration.

### 2.1. Qualitative Analysis of Main Compounds in Oleoresin

Qualitative analysis of oleoresin from *P. pinaster* was performed using GC-MS. As oleoresin contains terpenic compounds and resin acids, derivatization with diazomethane was conducted prior to analysis to convert the resin acids into their corresponding methyl ester derivatives. This approach enabled chromatographic analysis of both resin acids and more volatile terpenic compounds within a single run. A major advantage of diazomethane derivatization is its occurrence at room temperature, producing methyl ester derivatives with minimal isomerization. In contrast, other methylation techniques often require elevated temperatures, which may induce double-bond isomerization [34].

Of the several methods developed, Method 1 was chosen for the analysis of oleoresin from *P. pinaster*, because oleoresin includes both highly volatile compounds (monoterpenic compounds) and less volatile compounds (resin acids) and, therefore, the GC-MS analysis requires a broader temperature program to efficiently separate all the compounds in the sample (80–290 °C). Limonene and tetracosane were the internal standards (IS) employed in this analysis. Notably, limonene was absent from the oleoresin from *P. pinaster*, confirming its suitability as IS without interference from endogenous compounds.

**Figure 2:**
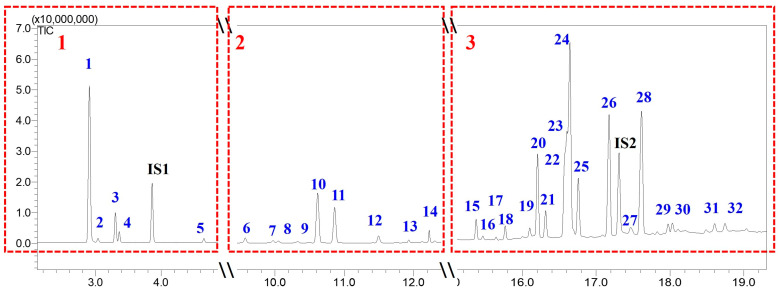
Example of GC-MS total ion chromatogram (TIC) of methylated oleoresin from *P. pinaster* using Method 1. Peaks are grouped into three regions: volatile monoterpenic compounds (**1**), intermediate volatility sesquiterpenic compounds (**2**), and diterpenic compounds (**3**). IS1: limonene. IS2: tetracosane.

After GC-MS analysis, the main compounds were identified by comparing their elution order, mass spectrum, and fragmentation patterns with Wiley (John Wiley & Sons, Inc. (Hoboken, NJ, USA) and U.S. National Institute of Standards and Technology (NIST) (Gaithersburg, MD, USA) database libraries (Wiley 226, NIST17-1, NIST17-2, and NIST17s), and literature data [3,19,20,35,36,37]. The results are presented in Figure 2 and Table S1.

As seen in Figure 2, the GC-MS total ion chromatogram (TIC) of methylated oleoresin from *P. pinaster* revealed a complex mixture of compounds, with three main elution regions. The chromatographic peaks of more volatile compounds (monoterpenic compounds) appear in the first region with retention time (RT) 2.90–4.65 min, while the second region (RT: 9.57–12.22 min) is populated by chromatographic peaks corresponding to compounds with intermediate volatility (sesquiterpenic compounds). The less volatile neutral diterpenic compounds and resin acid methyl esters appear in the region of RT 15.37–18.74 min. In total, 30 peaks are observed in the GC-MS chromatogram, leading to the identification of 32 different compounds (due to peak overlap among compounds **22**, **23**, and **24**) in the oleoresin from *P. pinaster*.

The first region in the GC-MS chromatogram is characterized by the elution of a group of five compounds with identical M^+^ at *m/z* 136 and with major fragment ions at *m/z* 121 and 93 corresponding to the loss of CH_3_ and C_3_H_7_, respectively [38]. The analysis of their chromatographic behavior and mass spectra allowed the identification of the five compounds as the following monoterpenic compounds: α-pinene (**1**), camphene (**2**), β-pinene (**3**), β-myrcene (**4**), and 3-carene (**5**) [3,19,20,34,35,36,37].

The second region of the GC-MS chromatogram was populated by a group of nine compounds with M^+^ at *m/z* 204, which were identified as the following sesquiterpenic compounds: longipinene (**6**), longicyclene (**7**), copaene (**8**), sativene (**9**), longifolene (**10**), β-caryophyllene (**11**), humulene (**12**), germacrene (**13**), and α-cubebene (**14**) [3,19,31,39,40,41,42,43]. The most abundant fragment ion for longifolene (**10**), germacrene (**13**), and α-cubebene (**14**) occur at *m/z* 161 ([M–15–28]) due to the loss of both CH_3_ and C_2_H_4_ [42]. The major fragment ion for longipinene (**6**) and copaene (**8**) was at *m/z* 119, which corresponds to the formation of a dimethyl tropylium cation. β-Caryophyllene (**11**) and humulene (**12**) show fragment ion at *m/z* 93 due to the formation of a dimethyl cyclopentadienyl cation. Sativene (**9**) shows its characteristic peak at *m/z* 108 and longicyclene (**7**) at *m/z* 94 [3,31,39,40,41,42,43].

The third region of the GC-MS chromatogram corresponds to the elution of the less volatile neutral diterpenic compounds and resin acid methyl esters. Several studies have described characteristic fragmentation patterns that allowed the identification of 18 compounds [19,29,30,43,44]. Based on that, compounds **15**–**19** were identified as neutral diterpenic compounds. Compounds **15** and **17**, both with M^+^ at *m/z* 290 and with major fragment ions at *m/z* 191 and 146, were identified as isoabienol and 11,13-labdien-8-ol, respectively. Compound **16** with M^+^ at *m/z* 288 and a major fragment ion at *m/z* 133 was identified as isopimarol [30,43,44]. Compounds **18** and **19**, both with M^+^ at *m/z* 286, were identified as pimaral and isopimaral, respectively, with characteristic fragment ions at *m/z* 271 ([M–15]) and 257 ([M–29]), corresponding to the loss of CH_3_ and C_2_H_5_, respectively [29,30].

**Figure 3:**
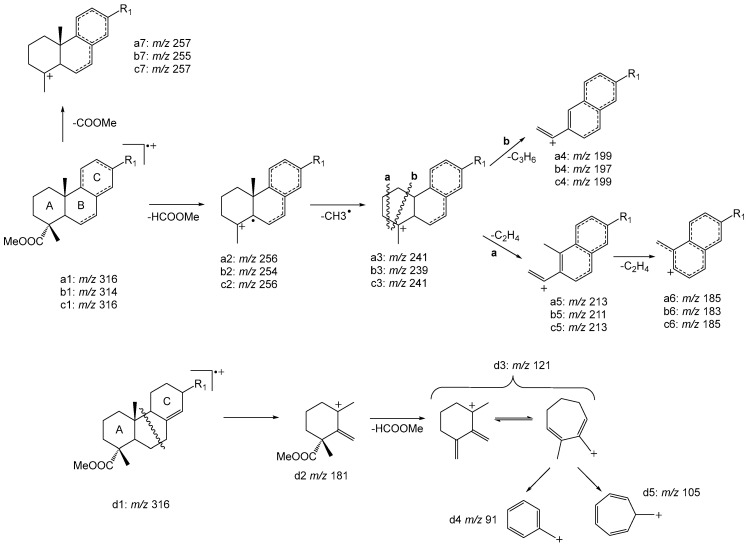
Possible fragmentation patterns of resin acids containing two unsaturations (a1–a7); three unsaturations (b1–b7) or two unsaturations on the same C ring (c1–c7); absence of unsaturation in B ring (d1–d5).

Among the pimaric-type resin acids, compounds **20**, **21**, and **22**, with M^+^ at *m/z* 316, were identified as pimaric, sandaracopimaric, and isopimaric acids, respectively [29,30]. Figure 3 provides a detailed overview of the proposed fragmentation patterns. All these compounds present major fragment ions at *m/z* 301 ([M–15]) and 257 ([M–59]) corresponding to the loss of CH_3_ and COOCH_3_ (fragment a7). Another very important fragment that allowed the differentiation among the pimaric-type resin acids is the fragment ion at *m/z* 121 (fragment d3) present only in the pimaric (**20**) and sandaracopimaric acids (**21**), which indicates the absence of a double bond in the B ring. In contrast, the base peak at *m/z* 241 (fragment a3) observed in the electron ionization mass spectrum (EI-MS) of compound **22** indicates one double bond in the B ring, corroborating the identification of this compound as isopimaric acid (Figure 3) [19,36].

Among the abietic-type resin acids, palustric (**23**), levopimaric (**24**), abietic (**26**), and neoabietic acids (**28**) were successfully identified as their methyl ester derivatives, with M^+^ at *m/z* 316 and main fragment ion at *m/z* 301 ([M–15]) related to the loss of CH_3_. Palustric acid (**23**) shows also a prominent peak at *m/z* 241 (fragment c3) which is related to the presence of two bonds in the C ring (Figure 3). Abietic acid (**26**) has its main peak fragment ions at *m/z* 256 (fragment a2) and 241 (fragment a3) related to the loss of HCOOCH_3_ and plus CH_3_, respectively. Moreover, the presence of a fragment ion at *m/z* 121 (fragment d3) allowed the identification of neoabietic acid (**28**) and the presence of both *m/z* 121 (fragment d3) and 146 ([M–170]) enabled the identification of levopimaric acid (**24**) [3,19,20,29,30,31,34,35,36,37].

Compound **25** was identified as dehydroabietic acid with M^+^ at *m/z* 314 (methyl ester) and a major fragment ion at *m/z* 239 (fragment b3), consistent with the fragmentation pathway shown in Figure 3, and indicative of the presence of three bonds on the same ring [19,41]. Compound **30** was identified as 7,13,15-abietatrienoic acid due to its molecular ion similar to dehydroabietic acid, however with a major fragment ion at *m/z* 254 (fragment b2) [43].

Compound **27** with M^+^ at *m/z* 316 was the only compound that could not be conclusively identified; however, based on its molecular mass and fragmentation pattern, it is likely an isomer of resin acids. Compound **29** with M^+^ at *m/z* 328 (methyl ester) and main fragment ion at *m/z* 253 (related to fragment b3) was identified as 7-oxodehydroabietic acid [19,36,41]. Finally, compounds **31** and **32** were identified as 15-hydroxydehydroabietic and 7-hydroxydehydroabietic acids, respectively, as they exhibited M^+^ at *m/z* 330 (methyl ester) with main fragment ions at 315 ([M–15]) and 255 ([M–75]) related to the loss of a CH_3_ plus the loss of HCOOCH_3_ (related to fragment b3), respectively (Figure 3) [3,19,20,29,30,31,34,35,36,37].

The main compounds identified in this study are consistent with previous reports on the chemical characterization of oleoresin by GC-MS [3,29,30,31,45]. For instance, Arrabal et al. showed that the dominant monoterpenic compounds in *P. pinaster* from Spain are α-pinene and β-pinene, and among the sesquiterpenic compounds, longifolene and β-caryophyllene are the most prominent. Among the resin acids, levopimaric, neoabietic, abietic, isopimaric, pimaric, and dehydroabietic acids were also identified after derivatization with TMAH [30]. However, these studies lack complete compound identification, as fragmentation patterns and RT are not reported, making reproducibility difficult. In contrast, our study provides a full characterization of oleoresin compounds. Moreover, the analytical approach described here not only confirms previously reported terpenic compounds and resin acids but also enables the detection of additional compounds such as longipinene, longicyclene, germacrene, 7-oxodehydroabietic acid, 15-hydroxydehydroabietic acid, and 7-hydroxydehydroabietic acid, thereby providing a more comprehensive chemical profile.

### 2.2. Quantitative Analysis of Main Compounds in Oleoresin

The quantification was performed using external calibration with the representative standards of α-pinene and abietic acid, employing limonene and tetracosane as IS, respectively. The selection of α-pinene as the representative calibration standard for all terpenic compounds and abietic acid for all resin acids was based on their structural similarity to the major constituents of each respective chemical family. Terpenic compounds share closely related hydrocarbon frameworks, leading to comparable ionization efficiencies and response factors under EI-MS, while resin acids possess similar diterpenic skeletons with comparable functional groups, allowing abietic acid to serve as a reliable representative standard. Calibration curves for both α-pinene and abietic acid showed excellent linearity (R^2^ > 0.997) across the tested mass ranges (Figures S1 and S2).

Table 1 illustrates the differences observed between relative quantification based on GC-MS peak area percentages and the more accurate quantification using the external calibration with α-pinene and abietic acid, for some of the terpenic compounds and resin acids constituents of oleoresin. The results demonstrate that relative quantification generally overestimates terpenic compound contents. For instance, α-pinene (**1**) accounted for 10.90 ± 2.60 w/w% in relative quantification but only 6.67 ± 1.08 w/w% with the quantification using external calibration, leading to a difference in total terpenic compound contents and in the total oleoresin content of 27.64 ± 3.20 and 17.98 ± 2.10 w/w%, respectively. An inverse trend was observed for the resin acids, for which external calibration yielded higher concentrations than those obtained by relative quantification. For example, abietic acid (**26**) showed a value of 12.60 ± 2.93 w/w% when quantified by external calibration, compared to 8.88 ± 1.33 w/w% determined by peak area percentage, resulting in total resin acids contents of 82.80 ± 6.20 and 64.69 ± 4.57 w/w%, respectively.

These discrepancies highlight the limitations of relying solely on peak area percentages, particularly when comparing different samples or analyzing complex matrices. By employing external calibration, the proposed method provides more accurate quantification, reduces systematic bias, and enables direct comparability across samples and studies. Notably, while relative quantification values are consistent with those reported by Arrabal et al. (30.20 w/w% terpenic compounds and 64.30 w/w% resin acids in *P. pinaster* after TMAH derivatization) [30], the present approach offers enhanced precision and reliability, which is critical for detailed chemical profiling of oleoresin.

Relative quantification is based solely on peak area percentages, which can only provide a relative proportion between detected compounds. As this approach does not account for non-eluted compounds, it cannot provide the entire sample mass. Furthermore, relative quantification often overestimates terpenic compounds due to their higher volatility, which leads to more intense chromatographic signals. In contrast, external calibration is far more accurate as it relies on accurate amounts of standards (α-pinene, abietic acid) and internal standards (limonene and tetracosane), showing an excellent linearity (R^2^ ≥ 0.997), enabling accurate information about the content of each compound in the pristine sample. If the total amount of quantified compounds does not account for the total mass of the sample, this would mean that the difference would come from non-eluted compounds, thus providing much more accurate information.

**Figure 4:**
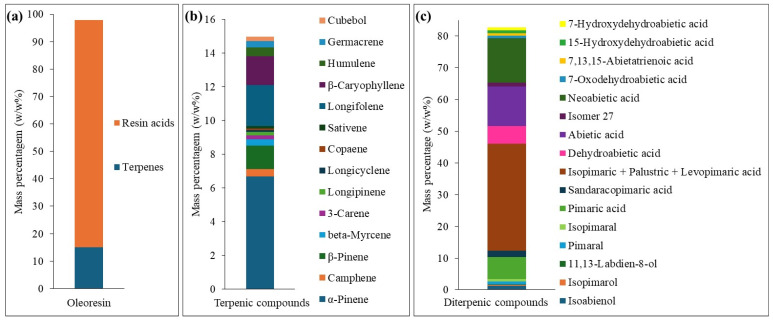
Quantification using external calibration, in mass percentage (w/w%) for (**a**) total of terpenic compounds and resin acids in oleoresin from *P. pinaster*; (**b**) identified terpenic compounds (mono- and sesquiterpenic compounds); (**c**) identified diterpenic compounds (neutral diterpenic compounds and resin acids). Values represent means (n = 3 independent derivatizations, each analyzed in triplicate).

Quantification of oleoresin components from *P. pinaster* using external calibration (Figure 4) reveals that mono- and sesquiterpenic compounds constitute 14.98 ± 2.10 w/w%, while neutral diterpenic compounds and resin acids account for 82.80 ± 6.20 w/w% (of which 3.45 ± 0.45 w/w% correspond to neutral diterpenic compounds), resulting in a total identified compounds content of 97.78 ± 6.57 w/w%. Among the mono- and sesquiterpenic compounds identified, α-pinene (**1**) is the major compound, accounting for 6.67 ± 1.08 w/w%, followed by longifolene (**10**), β-caryophyllene (**11**), and β-pinene (**3**) with 2.45 ± 0.20, 1.71 ± 0.15, and 1.40 ± 0.30 w/w%, respectively. Among the neutral diterpenic compounds, isoabienol is the major constituent, with 1.26 ± 0.20 w/w% of the total oleoresin mass. The predominant resin acids are neoabietic (**29**) and abietic acids (**26**) with 13.97 ± 1.70 and 12.60 ± 2.90 w/w% of the total oleoresin mass, respectively. Isopimaric (**22**), palustric (**23**), and levopimaric acids (**24**) were eluted with similar retention times (overlapping peaks from 16.57 to 16.64 min) and together accounted for a combined content of 33.75 ± 2.70 w/w%.

### 2.3. Qualitative Analysis of Main Resin Acids in Rosin

**Figure 5:**
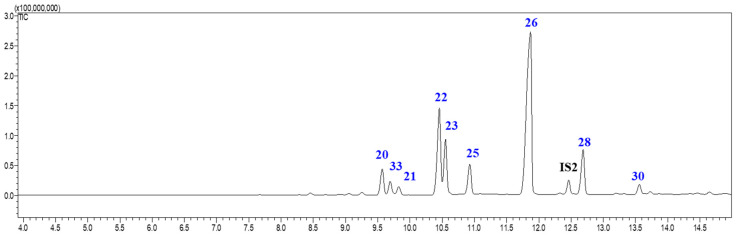
Example of GC-MS total ion chromatogram (TIC) of methylated *P. elliottii* rosin using Method 2. IS2: tetracosane.

To broaden the analytical scope of the proposed methodology, rosin samples were also analyzed (in the present study rosin from *P. elliottii* was selected due to its widespread industrial use and availability). The temperature program employed for rosin GC-MS analysis (Method 2) was distinct from that used for oleoresin (Method 1), due to the absence of the more volatile turpentine fraction and the comparable volatility of all resin acid methyl esters. Thus, the analysis could be initiated at 200 °C instead of 80 °C, enabling a shorter GC-MS run time. Tetracosane was used as the IS for rosin analysis. Figure 5 displays a representative rosin GC-MS total ion chromatogram (TIC), showing that the total GC-MS analysis time could be reduced from 19 min to less than 15 min, thereby facilitating faster sample evaluation.

The elution order, mass spectra analysis, and comparison with Wiley (John Wiley & Sons, Inc. (NJ, USA) and U.S. National Institute of Standards and Technology (NIST) (MD, USA) mass spectral libraries (Wiley 226, NIST17-1, NIST17-2, and NIST17s) and literature data allowed the successful identification of most compounds present in rosin from *P. elliottii* [3,29,30,31,45]. Moreover, the fragmentation patterns described in Figure 3 were also evaluated and led to the identification of 7 resin acids with similar M^+^ at *m/z* 316 (methyl ester). Among these, four pimaric-type resin acids were identified: pimaric (**20**), communic (**33**), sandaracopimaric (**21**), and isopimaric acids (**22**). A comparison with the resin acids present in the oleoresin of *P. pinaster* reveals the presence of an additional pimaric-type resin acid, the communic acid (**33**), which has been previously reported as exclusive in *P. elliottii* [29]. The abietic-type resin acids identified were palustric (**23**), abietic (**26**), and neoabietic acids (**28**). Two resin acids methyl esters with M^+^ at *m/z* 314 were identified as dehydroabietic (**25**) and 7,13,15-abietatrienoic acids (**30**) [19,41,43]. The main fragment ions observed for compounds **20**–**23**, **25**, **26**, **28**, and **30** are consistent with those presented in Table S1.

### 2.4. Quantitative Analysis of Main Resin Acids in Rosin

**Figure 6:**
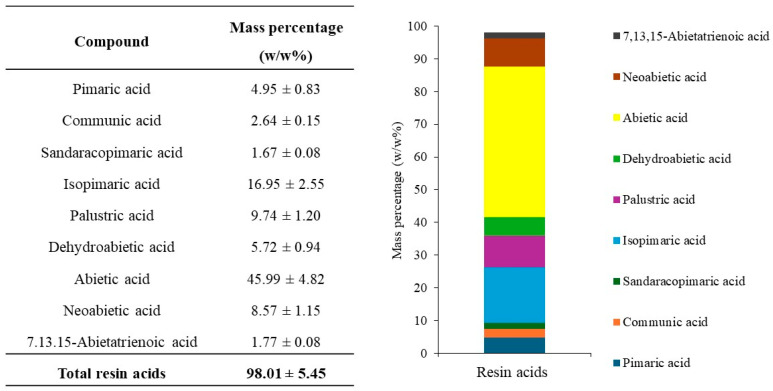
Quantification of resin acids in rosin from *P. elliottii* using external calibration of abietic acid, in mass percentage (w/w%). Values represent means ± standard deviation (n = 3 independent derivatizations, each analyzed in triplicate).

After the identification of the resin acids present in *P. elliottii* rosin, the quantitative analysis was performed using external calibration with abietic acid as a representative standard. Figure 6 shows that abietic acid (**26**) was the most abundant compound in this rosin sample, with 45.99 ± 4.82 w/w%, followed by isopimaric (**22**), palustric (**23**), and neoabietic acids (**28**) with 16.95 ± 2.55, 9.74 ± 1.20, and 8.57 ± 1.15 w/w%, respectively. The quantification of the identified resin acids accounted for 98.01 ± 5.45 w/w% of the total mass of analyzed rosin. This high percentage of identified compounds demonstrates the high reliability of the GC-MS method employed. Furthermore, derivatization with diazomethane was confirmed to be a highly effective approach as it resulted in a clean GC chromatogram, in contrast to previously reported methods, particularly when TMAH derivatization is used [29,30,31].

### 2.5. Qualitative Analysis of Main Terpenic Compounds in Turpentine

**Figure 7:**
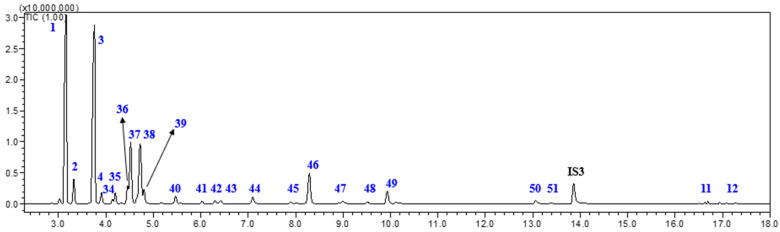
Example of GC-MS total ion chromatogram (TIC) of turpentine from *P. elliottii* using Method 3. IS3: carvacrol.

To further expand the analytical scope of the methodology, turpentine was also analyzed. In this study, turpentine from *P. elliottii* was selected due to its industrial relevance. The GC-MS temperature program for turpentine analysis (Method 3) differs from those used for oleoresin (Method 1) and rosin (Method 2), as turpentine does not require derivatization and contains more volatile components (mono- and sesquiterpenic compounds), which require a lower GC-MS temperature program. Furthermore, due to the high abundance of limonene and other terpenic compounds with similar RT (4.72 min) in *P. elliottii*, carvacrol was selected as an alternative IS for turpentine analysis from *P. elliottii*, since it shows a RT at 13.86 min when analyzed using Method 3 [3]. Figure 7 presents a representative GC-MS total ion chromatogram (TIC) obtained from this analysis.

The elution order, mass spectra analysis, fragmentation patterns, and comparison with Wiley (John Wiley & Sons, Inc. (NJ, USA) and U.S. National Institute of Standards and Technology (NIST) (MD, USA) MS spectra libraries (Wiley 226, NIST17-1, NIST17-2, and NIST17s) and literature data allowed the identification of most terpenic compounds present on turpentine from *P. elliottii*, which are listed in Table S2 [3,30,31,38,42]. Among them, 10 terpenic compounds with similar M^+^ at *m/z* 136 were identified as follows: α-pinene (**1**), camphene (**2**), β-pinene (**3**), β-myrcene (**4**), α-phellandrene (**35**), α-terpinene (**36**), _D_-limonene (**38**), γ-terpinene (**40**), 4-carene (**42**), and isoterpinolene (**43**). Compounds **34**, **37**, and **39** with M^+^ at *m/z* 138, 134, and 138 were identified as carane, *p*-cimene, and *p*-menth-3-ene, respectively. Compounds **41** and **45** with M^+^ at *m/z* 152 were identified as _L_-fenchone and camphor, respectively. Compounds **44**, **47**, **48**, and **49** with M^+^ at *m/z* 154 were identified as fenchol, borneol, _L_-4-terpineol, and α-terpineol, respectively. Compound **46** with a M^+^ at *m/z* 156 was identified as *o*-menthan-8-ol. Compounds **50**, **51**, **11**, and **12** with M^+^ at *m/z* 166, 196, 204, and 204 were identified as nopol, bornyl acetate, β-caryophyllene, and humulene, respectively.

The results obtained in this study are consistent with previous reports on turpentine composition, such as the one from Sadeghi et al., which identified similar terpenic compounds for turpentine from *P. eldarica* and *P. nigra* [40]. However, those authors identified only 10 terpenic compounds, whereas the present study identified 24 different terpenic compounds in a single GC-MS analysis (Table S2) [40].

The terpenic composition of turpentine from *P. elliottii* shows notable differences compared to that of oleoresin from *P. pinaster*. These variations may be attributed to pine species, geographic origin, and the distillation process used to separate turpentine from rosin, which can remove certain compounds or form others through chemical reactions. This underscores the importance of accurate compound identification, as their composition can vary significantly among samples.

### 2.6. Quantitative Analysis of Main Terpenic Compounds in Turpentine

**Figure 8:**
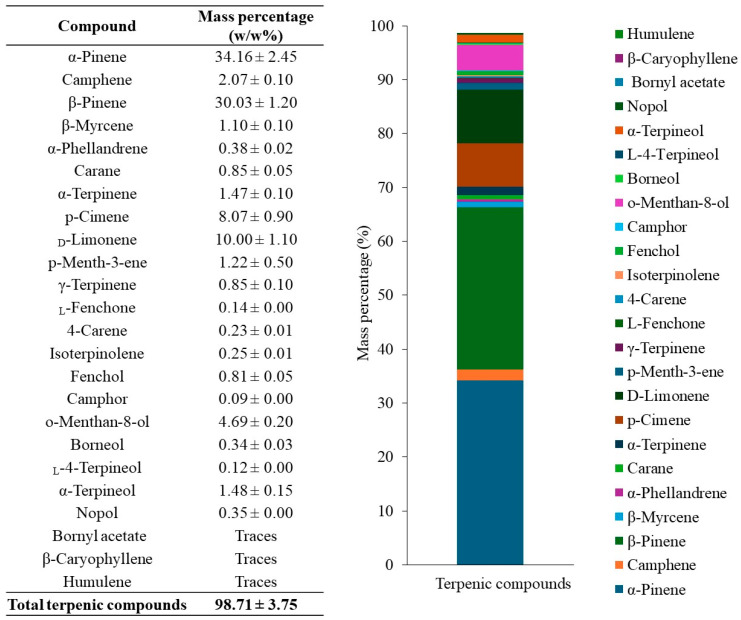
Quantification of terpenic compounds in turpentine from *P. elliottii* using an external calibration of α-pinene, in mass percentage (w/w%). Values represent means ± standard deviation (n = 3 independent derivatizations, each analyzed in triplicate).

After the identification of the 24 terpenic compounds in turpentine from *P. elliottii*, quantitative analysis was performed using external calibration with α-pinene as representative standard. Figure 8 shows that α-pinene (**1**) was the most abundant terpenic compound, accounting for 34.16 ± 2.45 w/w%, followed by β-pinene (**3**), _D_-limonene (**38**), and *p*-cimene (**37**) with 30.03 ± 1.20, 10.00 ± 1.10, and 8.07 ± 0.90 w/w%, respectively. Additional compounds present at mass percentages between 1.00 and 5.00 w/w% included camphene (**2**), β-myrcene (**4**), α-terpinene (**36**), *p*-menth-3-ene (**39**), *o*-menthan-8-ol (**46**), and α-terpineol (**49**). All remaining compounds showed mass percentages below 1.00 w/w%.

The quantification of identified terpenic compounds in turpentine from *P. elliottii* was nearly complete, reaching 98.71 ± 3.75 w/w% of the total mass of analyzed turpentine. Only three compounds were not quantified, demonstrating that the developed GC-MS method enables effective and rapid detection, identification, and quantification of low-abundance terpenic compounds in turpentine.

## 3. Materials and Methods

### 3.1. Materials

Oleoresin from *Pinus pinaster* Aiton (from a plantation at Gafanha da Nazaré, Aveiro, Portugal) was collected by the traditional tapping process using sulfuric acid as a stimulant paste. Rosin and turpentine from *Pinus elliottii var. elliottii* were kindly provided by PinoPine–Produtos Químicos S.A (Aveiro, Portugal). *N*-Methyl-*N*-(*p*-tolylsulfonyl)nitrosamide (purity 99%), tetracosane (purity 99%), abietic acid (purity 95%), limonene (purity 99%), carvacrol (purity 99%), and α-pinene (purity 98%) were obtained from Sigma-Aldrich (Sintra, Portugal). Dehydroabietic acid (purity 95%) was acquired from ChemPur (Karlsruhe, Germany). Common solvents and reagents, namely methanol, diethyl ether, dichloromethane, and potassium hydroxide were all of reagent grade and acquired from Sigma-Aldrich (Sintra, Portugal).

### 3.2. Gas Chromatography Coupled with Mass Spectrometry Analysis

#### 3.2.1. Derivatization by Methylation with Diazomethane

Before GC-MS analyses, oleoresin, rosin or abietic acid (external standard) were derivatized by methylation using diazomethane. Briefly, 20.0 mg of sample containing 0.4 mg of tetracosane, limonene, and/or carvacrol (internal standards, IS) were treated with diazomethane, generated from the reaction between a solution of *N*-methyl-*N*-(*p*-tolylsulfonyl)nitrosamide in diethyl ether (0.07 g/mL) with a methanolic solution of KOH (4.0 g/mL). The reaction was carried out at room temperature. To minimize the hazards associated with diazomethane, a tube-in-tube apparatus was used to avoid direct handling of the reagent, and all procedures were performed in a well-ventilated fume hood. After derivatization, excess solvent and diazomethane were removed at room temperature by purging the system with a nitrogen stream, which was passed through a trap containing acetic acid to safely neutralize any remaining diazomethane, thus providing a safer setup suitable for routine laboratory use. Diazomethane is a reagent that is widely known for being highly effective, and as it is used in excess (which is destroyed at the end of the reaction), it enables full conversion of the free acids. It is also known that under the mild conditions used here there are no reported side reactions [6,46,47].

#### 3.2.2. GC-MS Equipment

The derivatized oleoresin, rosin, or abietic acid was dissolved in 1.0 mL of dichloromethane. For the analysis of turpentine or α-pinene (external standard), 20.0 mg of sample was dissolved in 1.0 mL of dichloromethane. The GC–MS analysis was carried out using an ion quadrupole based GC–MS-QP2010 Ultra (Shimadzu, Kyoto, Japan) equipped with a capillary column DB-1 J&W (dimethylpolysiloxane, 30 m × 0.32 mm internal diameter, 0.25 μm thick, Santa Clara, CA, USA) and helium was used as the carrier gas (35 cm s^−1^). The mass spectrometer was operated in electron impact (EI) mode with energy of 70 eV, and the data were collected in a range of *m/z* 35–700 and Total Ion Chromatograms (TICs) are shown in this mass range. The ion source was kept at 250 °C. The split ratio was set to 1:50 [6].

#### 3.2.3. GC-MS Temperature Programs

Three temperature programs were optimized to match the volatility range of each pine matrix while keeping the GC-MS runs as short as possible. For oleoresin, we used a low initial temperature and a wider program to separate both monoterpenic compounds and resin acid methyl esters in a single run (Method 1). For rosin, which lacks the most volatile fraction, we started at a higher temperature and shortened the program, focusing on resin acids (Method 2). For turpentine, containing only mono and sesquiterpenic compounds, a shorter, lower temperature program was sufficient (Method 3). The methods are as follows:

Method 1: initial temperature 80 °C; 6 °C min^−1^ to 150 °C; 100 °C min^−1^ to 225 °C; 6 °C min^−1^ to 280 °C for 2 min. The injector and transfer line temperatures were 250 °C and 290 °C, respectively.

Method 2: initial temperature 200 °C for 2 min; 5 °C min^−1^ to 215 °C for 2 min; 5 °C min^−1^ to 250 °C for 1 min. The injector and transfer line temperatures were 250 °C and 290 °C, respectively.

Method 3: initial temperature 80 °C for 7 min; 5 °C min^−1^ to 120 °C; 30 °C min^−1^ to 180 °C for 3 min. The injector and transfer line temperatures were 250 °C and 290 °C, respectively.

#### 3.2.4. Qualitative Analysis

The compounds were identified by comparing their EI-MS fragmentation profiles with the Wiley (John Wiley & Sons, Inc. (NJ, USA) and U.S. National Institute of Standards and Technology (NIST) (MD, USA) mass spectral libraries (Wiley 226 and U.S. National Institute of Science and Technology (NIST17-1, NIST17-2, NIST17s)), as well as through comparison with the elution order and EI-MS spectra with literature data and authentic standard compounds (α-pinene, limonene, and abietic and dehydroabietic acids) [3,19,20,34,35,36,37].

#### 3.2.5. Quantitative Analysis

Quantitative analysis was performed using external calibration of α-pinene (for terpenes) and abietic acid (for resin acids) as representative standards. The calibration standards were prepared in 1.0 mL of dichloromethane containing 0.4 mg of IS (tetracosane, limonene, and/or carvacrol) over nine concentration levels spanning 0.017–17.0 mg and 0.25–15.0 mg for α-pinene and abietic acid, respectively, and analyzed in triplicate. Calibration curves were determined by plotting the peak area ratio (y) (defined as the peak area of each standard compound divided by the peak area of the IS) versus the mass of the standard compound (x). The mass-response relationship of the present method was required to be linear with R^2^ values ≥ 0.997.

### 3.3. Statistical Analysis

All GC-MS analyses were conducted in triplicate (*n* = 3 independent derivatizations, each analyzed in triplicate), and the results are expressed as mean values with their corresponding standard deviation (SD).

## 4. Conclusions

This study establishes three GC-MS methods using diazomethane derivatization and a single nonpolar DB-1 column with tailored temperature programs (Method 1: oleoresin; Method 2: rosin; Method 3: turpentine) for the simultaneous qualitative and quantitative analysis of the main compounds in oleoresin, turpentine, and rosin from different *Pinus* spp. The implementation of accurate quantification through external calibration employing structurally similar compounds as representative standards (α-pinene for terpenic compounds and abietic acid for resin acids) enabled the quantification of more than 95% of compounds across these chemically diverse pine matrices, thereby overcoming limitations of relative peak-area methods. These methods offer industrial laboratories a unified platform requiring only temperature program adjustments when switching between oleoresin, rosin, and turpentine analyses, thus enabling rapid quality control across production feedstocks. Furthermore, once GC-MS identification has been established, routine GC-FID quantification can be readily implemented, supporting high-throughput industrial process monitoring. In practice, this unified rapid GC-MS framework can be applied in resin-processing industries for routine quality control, in forestry programs for screening pine chemotypes, and in research laboratories for fast and reproducible chemical analysis. Overall, it offers a practical tool for laboratories that routinely analyze large series of pine-derived samples.

## Figures and Tables

**Table 1:** Comparison of the quantification using external calibration and peak area for selected terpenic compounds and resin acids in oleoresin from *P. pinaster*.

Compound	Mass Percentage (w/w%)	
	External Calibration	Peak Area Percentage
Mono- and sesquiterpenic compounds		
α-Pinene (**1**)	6.67 ± 1.08	10.90 ± 2.60
β-Pinene (**3**)	1.40 ± 0.30	1.70 ± 0.65
Longifolene (**10**)	2.45 ± 0.20	4.00 ± 1.34
β-Caryophyllene (**11**)	1.71 ± 0.15	2.80 ± 0.84
Total mono- and sesquiterpenic compounds *	14.98 ± 2.10	27.64 ± 3.20
Resin Acids		
Isopimaric + Palustric + Levopimaric acids (**22**, **23**, **24**)	33.75 ± 2.73	24.69 ± 3.10
Dehydroabietic acid (**25**)	5.45 ± 1.01	3.86 ± 0.80
Abietic acid (**26**)	12.60 ± 2.93	8.88 ± 1.33
Neoabietic acid (**29**)	13.97 ± 1.70	9.93 ± 1.10
Total resin acids *	82.80 ± 6.20	64.69 ± 4.57
Total *	97.28 ± 6.57	92.33 ± 7.20

Values represent means ± standard deviation (*n* = 3 independent derivatizations, each analyzed in triplicate). * Identified compounds.

## Data Availability

The data that support the findings of this study are available from the corresponding author upon reasonable request.

## Supplementary Materials

The following supporting information can be downloaded at https://www.mdpi.com/article/10.3390/ijms27041690/s1.

## Abbreviations

The following abbreviations are used in this manuscript:

EI-MS	Electron ionization mass spectrometry
GC-MS	Gas chromatography coupled with mass spectrometry
GC-FID	Gas chromatography coupled with flame ionization detector
IS	Internal standard
RT	Retention time
SD	Standard deviation
TMAH	Tetramethylammonium hydroxide
