# Supplementary material for: Rapid GC-MS Characterization of Oleoresin, Turpentine and Rosin Using Tailored Chromatographic Programs
Source: Int J Mol Sci. 2026 Feb 9;27(4):1690. doi: 10.3390/ijms27041690 (PMC12940598; doi:10.3390/ijms27041690)
Supplement: Supplementary file 1 [file ijms-27-01690-s001.zip › ijms-4120838-supplementary.pdf]

Supplementary Materials

Rapid GC-MS Characterization of Oleoresin, Turpentine, and Rosin Using Tailored Chromatographic Programs

Nalin Seixas [1*], Sónia A. O. Santos [1] and Armando J. D. Silvestre [1]

[1] CICECO - Aveiro Institute of Materials, Department of Chemistry, University of Aveiro, 3810-193 Aveiro, Portugal. nalinseixas@ua.pt (N.S), santos.sonia@ua.pt (S.A.O.S.), armsil@ua.pt (A.J.D.S.).

* Correspondence: nalinseixas@ua.pt

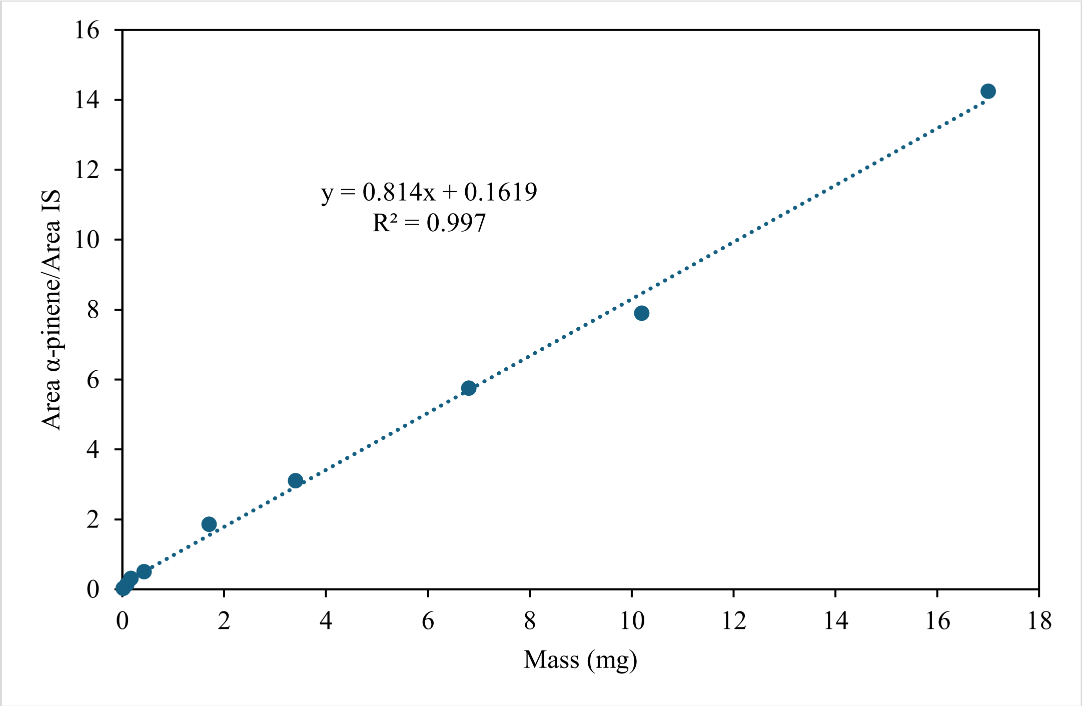

Figure S1. Calibration curve of α-pinene.

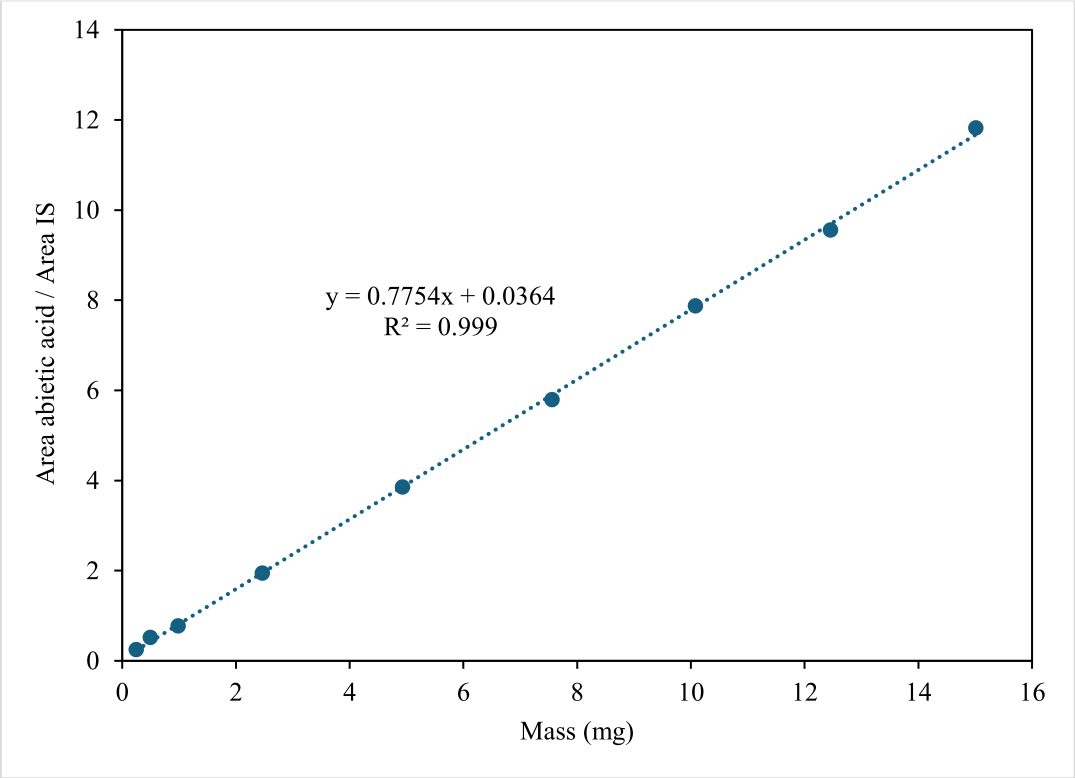

Figure S2. Calibration curves of abietic acid.

Table S1. Retention times and mass spectra data for the main compounds identified in oleoresin from *Pinus pinaster*.

Compound	RT (min)	Compound Identification	M^+	Fragment Ions
				m/z* intensity (%)
**Region 1**				
**1**	2.90	α-Pinene	136(25)	121(70), 105(25), 93(100), 92(80), 91(75), 79(30), 77(40)
**2**	3.03	Camphene	136(10)	121(70), 107(25), 93(100), 91(35), 79(40), 67(25)
**3**	3.30	β-Pinene	136(7)	121(15), 93(100), 91(30), 79(22), 77(20), 69(30), 41(25)
**4**	3.36	β-Myrcene	136(2)	93(100), 91(30), 79(18), 77(15), 69(70), 41(65)
**5**	4.65	3-Carene	136(75)	121(80), 105(25), 93(100), 91(60), 79(45), 77(30), 41(15)
**Region 2**				
**6**	9.57	Longipinene	204(10)	133(40), 119(100), 105(50), 93(35), 91(30), 79(17), 77(15), 69(15), 55(20), 41(15)
**7**	9.97	Longicyclene	204(25)	189(30), 161(50), 133(55), 119(80), 105(95), 94(100), 91(70), 79(45), 69(30), 55(30), 41(30)
**8**	10.04	Copaene	204(18)	161(90), 133(20), 119(100), 105(95), 93(50), 91(45), 81(30), 77(15), 55(15)
**9**	10.32	Sativene	204(18)	189(20), 161(60), 133(55), 119(50), 108(100), 105(65), 93(70), 91(60), 79(30), 55(20), 44(20), 41(20)
**10**	10.61	Longifolene	204(30)	189(50), 161(100), 133(60), 119(55), 107(73), 105(75), 94(75), 93(75), 91(75), 79(60), 67(30), 55(30), 41(25)
**11**	10.86	β-Caryophyllene	204(3)	189(20), 161(30), 147(30), 133(95), 120(50), 105(60), 93(100), 79(60), 69(65), 55(30), 41(50)
**12**	11.49	Humulene	204(3)	147(20), 121(25), 107(15), 93(100), 80(30), 69(15), 55(10)
**13**	11.93	Germacrene	204(12)	161(100), 133(35), 119(60), 105(80), 91(70), 81(50), 79(35), 77(25), 69(15), 67(15), 55(20), 41(20)
**14**	12.22	α-Cubebene	204(30)	189(15), 161(100), 147(15), 133(35), 119(55), 105(85), 93(60), 81(50), 69(25), 55(30), 43(50), 41(25)
**Region 3**				

**15**	15.37	Isoabienol	290(2)	257(20), 191(100), 177(30), 149(20), 135(20), 121(35), 109(40), 95(60), 81(55), 69(50), 55(35), 43(30)
**16**	15.46	Isopimarol	288(5)	272(25), 257(98), 207(25), 173(25), 159(20), 147(40), 133(100), 119(70), 107(55), 93(55), 91(70), 81(35), 79(40), 55(35)
**17**	15.64	11,13-Labdien-8-ol	290(2)	257(20), 187(50), 146(100), 133(60), 131(35), 123(25), 117(25), 109(25), 101(45), 92(45), 91(40), 81(20)
**18**	15.76	Pimaral	286(15)	271(45), 257(95), 207(25), 187(30), 173(30), 161(40), 146(60), 135(55), 133(95), 123(100), 107(85), 91(85), 81(85), 67(50), 55(45)
**19**	16.10	Isopimaral	286(20)	257(50), 243(25), 229(23), 201(27), 187(98), 173(25), 159(25), 145(70), 131(100), 119(60), 105(80), 91(70), 81(70), 67(30), 55(45)
**20**	16.20	Pimaric acid**	316(5)	257(15), 180(18), 133(14), 121(100)
**21**	16.31	Sandaracopimaric acid**	316(6)	257(35), 133(20), 121(100), 107(20), 93(20), 91(18), 81(15)
**22**	16.57	Isopimaric acid**	316(18)	301(25), 257(40), 241(100), 227(20), 201(20), 187(30), 133(25), 121(30), 105(35), 91(25)
**23**	16.60	Palustric acid**	316(65)	301(95), 257(20), 241(100),213(20), 185(30), 159(35), 149(40), 133(25), 105(30), 91(20)
**24**	16.64	Levopimaric acid**	316(60)	187(35), 146(100), 133(45), 121(95), 117(20), 109(25), 105(30), 101(35), 92(75), 91(70)
**25**	16.75	Dehydroabietic acid**	314(10)	299(12), 255(5), 239(100)
**26**	17.17	Abietic acid**	316(50)	273(20), 256(100), 241(80), 213(60), 185(45), 131(30), 121(35), 105(25), 91(20)
**27**	17.47	Isomer of abietic-type resin acid**	316(25)	301(55), 257(75), 241(50), 207(60), 173(55), 133(65), 121(100)
**28**	17.61	Neoabietic acid**	316(30)	148(30), 135(100), 121(35)
**29**	17.97	7-Oxodehydroabietic acid**	328(30)	369(20), 253(100), 213(25), 187(35)
**30**	18.03	7,13,15-Abietatrienoic acid**	314(50)	255(30), 254(100), 239(55), 185(20), 143(18), 134(30), 131(30), 121(50), 119(20), 117(25), 107(20), 105(23), 91(30)
**31**	18.60	15-Hydroxydehydroabietic acid**	330(10)	315(90), 255(100), 237(45), 197(20), 109(25)
**32**	18.74	7-Hydroxydehydroabietic acid**	330(25)	315(15), 255(100), 239(25), 207(40), 146(35), 121(40), 109(25), 91(25)

*In the case of product ions, only those with relative intensity above 15.0% are reported. **Identified as methyl ester derivatives.

Table S2. Retention times and mass spectra data for the main compounds identified in turpentine from *Pinus elliottii*.

**Compound**	**RT (min)**	**Compound Identification**	**M⁺**	**Fragment Ions**
				m/z* intensity (%)
**1**	3.16	α-Pinene	136(10)	121(18), 105(15), 93(100), 92(42), 91(40), 77(30)
**2**	3.32	Camphene	136(10)	121(75), 107(30), 93(100), 91(20), 79(45), 67(25)
**3**	3.75	β-Pinene	136(10)	121(15), 93(100), 91(25), 79(22), 77(20), 69(32), 41(30)
**4**	3.90	β-Myrcene	136(2)	93(100), 91(25), 79(20), 77(18), 69(72), 41(70)
**34**	4.14	4-Carane	138(18)	123(22), 109(15), 95(100), 81(50), 67(60), 55(30), 41(22)
**35**	4.20	α-Phellandrene	136(22)	93(100), 92(30), 91(52), 77(40)
**36**	4.45	α-Terpinene	136(50)	121(100), 105(25), 93(90), 91(42), 79(30), 77(30)
**37**	4.52	*p*-Cimene	134(30)	119(100), 117(15), 91(23)
**38**	4.72	D-Limonene	136(12)	121(25), 107(25), 93(75), 79(25), 68(100), 53(20), 41(20)
**39**	4.80	*p*-Menth-3-ene	138(40)	123(28), 95(100), 81(80), 67(52), 55(25), 41(22)
**40**	5.47	γ-Terpinene	136(32)	121(30), 93(100), 91(50), 77(35), 43(15)
**41**	6.02	L-Fenchone	152(12)	81(100), 69(45)
**42**	6.30	4-Carene	136(45)	121(100), 105(25), 93(80), 91(38), 79(40), 77(25)
**43**	6.42	Isoterpinolene	136(73)	121(90), 93(100), 91(50), 79(45), 77(35)
**44**	7.10	Fenchol	154(2)	111(15), 93(15), 84(20), 81(100), 80(60), 69(20), 55(18), 43(25)
**45**	7.89	Camphor	152(22)	108(50), 95(100), 81(90), 69(40), 55(40), 41(32)
**46**	8.29	*o*-Menthan-8-ol	156(2)	59(100)

**47**	8.99	Borneol	154(2)	110(22), 95(100)
**48**	9.51	L-4-Terpineol	154(10)	136(15), 111(58), 93(55), 71(100), 69(20), 55(20), 43(35)
**49**	9.93	α-Terpineol	154(2)	136(45), 121(65), 93(75), 81(50), 67(20), 59(100)
**50**	13.06	Nopol	166(4)	105(100), 91(22), 79(28)
**51**	13.38	Bornyl acetate	196(2)	136(30), 121(50), 95(100), 93(50), 80(25), 43(50)
**11**	16.69	β-Caryophyllene	204(5)	189(15), 161(25), 147(22), 133(80), 119(50), 105(82), 93(100), 91(95), 79(75), 69(80), 41(55)
**12**	17.08	Humulene	204(5)	147(20), 121(28), 107(25), 93(100), 80(30), 44(25)

*In the case of product ions, only those with relative intensity above 15.0% are reported.
